# Multicomponent, high-intensity, and patient-centered care intervention for complex patients in transitional care: SPICA program

**DOI:** 10.3389/fmed.2022.1033689

**Published:** 2022-11-24

**Authors:** Miguel García-Hernández, Beatriz González de León, Silvia Barreto-Cruz, José Ramón Vázquez-Díaz

**Affiliations:** ^1^Unidad Docente de Atención Familiar y Comunitaria La Laguna-Tenerife Norte, Gerencia de Atención Primaria del Área de Salud de Tenerife, Santa Cruz de Tenerife, Spain; ^2^Network for Research on Chronicity, Primary Care, and Health Promotion (RICAPPS), Tenerife, Spain

**Keywords:** family practice, multimorbidity, internship and residency, patient care management, patient-centered care, patient discharge, primary health care, transitional care

## Abstract

Multimorbidity is increasingly present in our environment. Besides, this is accompanied by a deterioration of social and environmental conditions and affects the self-care ability and access to health resources, worsening health outcomes and determining a greater complexity of care. Different multidisciplinary and multicomponent programs have been proposed for the care of complex patients around hospital discharge, and patient-centered coordination models may lead to better results than the traditional ones for this type of patient. However, programs with these characteristics have not been systematically implemented in our country, despite the positive results obtained. Hospital Universitario de Canarias cares for patients from the northern area of Tenerife and La Palma, Spain. In this hospital, a multicomponent and high-intensity care program is carried out by a multidisciplinary team (made up of family doctors and nurses together with social workers) with complex patients in the transition of care (SPICA program). The aim of this program is to guarantee social and family reintegration and improve the continuity of primary healthcare for discharged patients, following the patient-centered clinical method. Implementing multidisciplinary and high-intensity programs would improve clinical outcomes and would be cost-effective. This kind of program is directly related to the current clinical governance directions. In addition, as the SPICA program is integrated into a Family and Community Care Teaching Unit for the training of both specialist doctors and specialist nurses, it becomes a place where the specific methodology of those specialties can be carried out in transitional care. During these 22 years of implementation, its continuous quality management system has allowed it to generate an important learning curve and incorporate constant improvements in its work processes and procedures. Currently, research projects are planned to reevaluate the effectiveness of individualized care plans and the cost-effectiveness of the program.

## Introduction

### Chronic conditions and multimorbidity

Chronic diseases are becoming more frequent. They are considered to be responsible for 74% of the deaths in the world (1) and, according to the Global Burden of Disease and Injury in 2019, account for more than 80% of disability-adjusted life years in Europe (2). Moreover, analyses carried out in Spain have postulated that the use of primary care increases in people with chronic diseases (3–5).

Although there are many definitions, multimorbidity is mainly considered to be the presence of more than one chronic condition, which are clinical and non-clinical (6). The presence of multimorbidity is increasingly present worldwide (7). It has been observed in studies with Spanish patients that those who use primary care the most are multimorbid people (3, 4). This supposes significant challenges for health systems (8, 9). It has been considered to increase mortality, decrease quality of life (10, 11), and increase costs and negative consequences for patients (12, 13).

Associated with the concept of multimorbidity, we found the concept of complexity. The presence of several chronic diseases is added to the fact that there are social and environmental conditions that have an impact on self-care and access to resources (14). In other words, not only the number of simultaneous chronic diseases influences health but also the severity of them added to several psychosocial factors (15). Complexity could be understood as a dynamic state in which socioeconomic, cultural, environmental, behavioral, and biological aspects operate as factors that hinder the understanding and management of health in certain people (16). Consequently, the interrelation between different factors may lead to interactions of diagnostic procedures or interventions, different treatment strategies, and multiple healthcare (16). That is why we distinguished between the complexity of the case, which refers to overlapping diseases and symptoms, and the complexity of care, which refers to the provision of healthcare and the joint involvement of systems and specialties. A complex case does not necessarily need complex care and *vice versa* despite, in most cases, both appear. In addition, complexity in terms of healthcare utilization can be identified and may manifest as care-regimen complexity, healthcare system navigation challenges, or complex networks of healthcare providers (17). For that reason, we must take into account all types of complexity when approaching a patient. If we did not, medical care would not be efficient (18).

### Complexity

Thus, complexity, the quality of self-care, and consequently health outcomes depend on the balance between the patient/relatives' capacity (skills, resources, or willingness to address the demands, including physical and psychological functioning, socioeconomic resources, social support, literacy, attitudes, and beliefs) and the workload of the demands (all the tasks and responsibilities that people face on a day-to-day basis) (19). Given this perspective, it is clear that the disease-centered care model cannot be applied to complex patients (20). The need to care for this kind of patient has led experts to seek a change in health systems with perspectives of systemic thinking and the search for common goals (21).

### Care transition and transitional patient care

Among the different elements that increase patients' complexity, the care transition processes between levels of healthcare should be highlighted. Patients face several difficulties with healthcare system navigation, so the transition of care from home to the hospital and back to home after discharge has a great impact on patients. In the case of complex patients, there is a high susceptibility to suffering an interruption in the continuity of care (22, 23). This lack of continuity has not only been perceived by professionals but also by patients. Several experiences of patients in different countries point to the presence of coordination gaps between levels. Patients perceive that information is often not passed on during transitions from hospitals to community settings after discharge (24). In fact, deficits in communication and information transfer at hospital discharge are common and may adversely affect patient care, in terms of clinical outcomes, risk of readmission, and quality of care and costs (23, 25).

Likewise, some reasons could explain this discontinuation. First, the physical remoteness of family doctors hinders follow-up on patients admitted to the hospital. Although the new technological and information tools have improved the transmission of information between levels, they fail to replace the benefit of direct contact between professionals and patients. Regarding the organization, the care objectives of family doctors and hospital specialists may not be shared, which can cause coordination problems. Likewise, the patient-centered clinical methods of family doctors, a core value in family medicine (26, 27), contrast from those of hospital doctors, which are centered on the disease. That determines the difference between actions and decisions. Finally, complex patients, due to their inherent characteristics, have a lower capacity to face by their own means the necessary care that originates after hospital admission (28).

There are several strategies that have been carried out with the intention of overcoming this complexity and improving health outcomes, especially in transitional care. Some of these strategies demonstrate a reduction in hospital readmissions and the average stay (29–31). Among them, it has been observed that uniprofessional interventions do not show significant results on aspects such as hospital readmissions (32) and that it could be more appropriate to design multiprofessional and comprehensive care programs in which the patient is assessed and treated as a whole, and not just as the sum of its diagnostics (33, 34). Additionally, in the context of transitional care, high-intensity interventions have been defined as those long-term interventions committed to continuity of care, those which involve patient and caregivers, and those performed before, during, and after hospital discharge (35); and multicomponent interventions are those consisting of at least two simple components. It has been observed that high-intensity, multicomponent, and multidisciplinary interventions are likely to be effective in reducing readmission rates (33, 35, 36). Besides, mortality and quality of life improved with high-intensity and complexity (i.e., frequent contacts and more intervention components) of transitional care interventions (37).

In contrast, we cannot ignore the need for the patient to take an active part in this process. In this regard, we have the so-called patient-centered care (PCC) (38–40). Person-centered interventions during the care transition of complex patients have been identified by patients and relatives as facilitating factors of healthcare (41). So, patient involvement in care planning increases adherence to the care plan and improves the quality of life (42). This leads us to shared decision-making, which is a part of PCC. However, associated with the presence of great complexity of management, these patients find themselves in a stressful life situation, which makes the shared decision-making process especially complicated.

A multicomponent and high-intensity care program, using the PCC method, is carried out by a multidisciplinary team with complex patients in the transition of care, which is named as SPICA program. The main aim is to guarantee the continuity of care for hospitalized patients and improve their socio-family reintegration of them after hospital discharge.

## Context (setting and population) in which the innovation occurs

SPICA is a technical name formed by the acronym for “Subprograma de Integración y Coordinación Asistencial” (care integration and coordination subprogram -or subprocess, too-), but “Spica” also is a Latin word which means both spike and tenon. As is well known, a spike is an inflorescence formed by a set of wheat grains that are arranged along an axis, which holds them together. Furthermore, a tenon is a piece widely used in carpentry to join two elements and makes an invisible junction. Therefore, the SPICA program can be considered metaphorically as that tenon that brings together all the elements to maintain the continuity of transitional care. Furthermore, it does it discreetly, without making these unifying elements too visible. SPICA program serves as a link between the patient, primary care, and the hospital providers to maintain continuity of care (43).

SPICA is identified as an integrated health service delivery in which the patient is the subject of the integration of different elements, which are needed to facilitate their care. From the point of view of integration typologies, SPICA incorporates the elements that have been described (system, organizational, functional, professional, service, and personal), with different degrees of intensity (44). In turn, it is a model that encompasses the individual integrated care that includes case management, individual care planning, and patient-centered medical home coordination. Being developed in the care transition, the program focuses on those hospitalized patients who present greater complexity, and therefore, greater difficulty and support needs to return home.

Its activity takes place at the Teaching Unit of Family and Community Care “La Laguna-Tenerife Norte” (45), which is located in Hospital Universitario de Canarias and attends to patients admitted to it, with a reference population of ~384,000 people, of which 338,000 are adults.

This program is currently made up of 5 teams, and each one is made up of a family doctor and a primary care nurse. It also has administrative support. It is coordinated by one of the team's family doctors. In turn, this coordinator reports to the director of this Teaching Unit.

The team has management by objectives and values, linked to incentives, with quantitative and qualitative components.

The base of patients of which SPICA works is around 22.000 hospital admissions (2021) in Hospital Universitario de Canarias (La Laguna), of which 16.300 constitute the actual target population for Spica (patients discharged from medical services or surgeries and more than 1 day of hospital stay).

Thus, from a quantitative point of view, the overall objective of this program is to include 900 patients per year with established quality standards (representing at least 5.5% of target patients). An efficiency of 85% is required from the teams, that is, out of these 900 patients, at least 792 (12%) must be discharged home (which represents at least 4.9% of hospital discharges). The qualitative objectives are related to the content and timing of the global care plan. This plan must be available before the first appointment with primary care professionals after discharge.

The program works with highly complex patients without specific age criteria or reason for admission if they meet the inclusion criteria established by the program itself (refer to Table 1). The inclusion criteria have been selected in relation to the characteristics of the patients that determine a complex hospitalization or a greater possibility of difficulties or barriers at discharge.

**Table 1:** Inclusion criteria for the SPICA program.

**Inclusion criteria (at least, 1 major or 3 minor criteria)**	
**Major criteria**	**Minor criteria**
• Patient receiving home care *(confined/immobilized)*. • Terminal disease (end of life care). • Cognitive impairment (Pfeiffer test >4). • Dependency in activities of daily living (Katz index > A). • Disabling bone fracture. • Living alone. • Previously attended by SPICA program.	• Age over 74. • >2 Chronic diseases (i.e., diabetes mellitus, heart failure, renal chronic disease, Parkinson's disease, COPD or cirrhosis). • Poor self-perceived health. • Major depression. • Severe visual or hearing impairment. • Malnutrition. • Hospitalization in the last 6 months. • Dependency in instrumental activities of daily living. • Falls in the last 3 months.

Patients access the program through the following two different ways:

(A) Hospital inpatient screening: The program considers the group of hospitalized patients as a population with risk components, especially in terms of continuity of care after discharge. For this reason, the screening process is carried out by the team's own professionals, who include the patients they consider to benefit most from their care (according to the criteria. Refer to Table 1).

(B) Opportunistic recruitment: It is carried out at the request of the service responsible for the hospital admission, primary care doctor, family, social workers, or the own patient.

As the SPICA program is part of the Canary Health Service, it will not be able to coordinate those patients who are referred from or to private health services. Likewise, when patients are discharged prematurely or the hospitalization time is too short (72 h), they cannot be coordinated. Finally, those patients who reject it or who are transferred to other hospitals are also excluded.

As mentioned earlier, although SPICA attends without age restriction, hospital inpatient screening among pediatric, obstetric, and psychiatric patients is not performed, and only opportunistic recruitment is conducted.

## Detail to understand key programmatic elements

Since its design, the SPICA program incorporated and developed the core elements of the Chronic Care Model (46–49). SPICA professionals work in functional alliance with other medical specialists (both medical and surgical specialties), social workers and nurses specialized in other areas (depending on the case they attend), and family doctors and primary healthcare nurses. So, SPICA is a patient-centered program that incorporates comprehensive and contextual assessment, evidence-based clinical practice, and intra- and inter-level and inter-sectorial (with social services) coordination. In addition, the program purposes problem-solving through a multiprofessional and cooperative style.

As it has been said, this is a high-intensity and multicomponent program. So, it was ahead of its time, since it was not until a few years after its design that the efficacy of high-intensity and multicomponent interventions was confirmed (33, 35, 37).

### Patient-centered comprehensive assessment

SPICA program intends to move away from transversality in care, and its main aim is to maintain continuity of care. When a patient is included in the program, and before the first assessment, SPICA professionals inquire on patients' history, their pathobiography, and context in which they live before admission. In this way, professionals seek a unique integrated understanding of each patient and acquire longitudinal knowledge of them. Deep knowledge of the previous state of the patient allows us to take the helm from primary care, continue it during hospitalization, and return it back to primary care after discharge.

After acquiring this prior information, the team performs its face-to-face evaluation of the patient. The patient assessment includes a comprehensive biopsychosocial evaluation (including patients, their family environment, and the available resources) (refer to Table 2 and more detailed information in Supplementary Appendix I) and is complementary and synergistic to the clinical evaluation carried out in the hospital. In this part, the objective is to know not only the patient's disease (medical history, physical examinations, and diagnostic tests) and experience of the disease but also their aspirations and their meaning. Specifically, illness experience exploration and the four key dimensions of it (feelings, ideas, functions, and expectations) (39, 40) are the essential activities of the SPICA program that is always performed. Besides, the program pursues understanding the social context in which the patients live their lives. Families and their life cycles and concerns are taken into account and displayed by structural and functional genogram, which is routinely performed on every patient. Thus, close contact is maintained with the patient's relatives, and family interventions are habitually conducted. This allows the professional to reach a proper meaning of the problems and attend to patients' perceptions of health and experience of the disease. In stressful circumstances such as hospital admission, changes occur in the elements; therefore, the understanding of the problems can change, and this leads to a permanent construction and reconstruction of the meaning. Bearing in mind this dynamic condition of construction and reconstruction of meanings is what allows us to formulate all the patient's problems before discharge. Therefore, the evaluation pursues an intentionality that is not merely contemplative but operational, with an aim at seeking keys to act and establish an adequate helping relationship (50).

**Table 2:** SPICA program comprehensive assessment template.

**1. Pre-assessment data**	
1	Identification data
2	Cause of hospital admission and responsible clinical service
3	Personal medical history
4	Active medical problems before to admission
5	Pharmacological and non-pharmacological treatment before to admission
**2. Comprehensive assessment**	
1	Cognitive assessment
2	Functional assessment
3	Psychoaffective assessment
4	Self-perceived health, Illness experience, and treatment experience.
5	Hearing assessment
6	Visual assessment
7	Nutritional assessment
8	Urinary and fecal elimination assessment
9	Condition of the skin
10	Fall risk assessment
11	Family assessment: Functional and structural genogram, family apgar, caregiver, etc
12	Patient economic resources
13	Patient material resources
14	Home assessment through an interview (if it is necessary, a home visit from the social worker is requested)

### Helping relationship: Patients, families, and professionals

During hospital admission, an increase in the complexity of future care is often derived, since the burdens of illness and care tend to raise and the ability to cope with it decreases (19). One of the challenges is to intervene to reduce these burdens of disease and/or care and increase the capacities and/or possibilities of patients to assume them. For that reason, the SPICA team has the role of carrying out horizontal coordination with all the professionals who care for the patient during hospitalization. Moreover, it also has the function of performing vertical coordination with primary care professionals, other professionals outside the hospital environment, the family/caregiver, the social network, and the patient.

As the SPICA program works with those patients who present not only the complexity of the case but also the complexity of care, in many cases, a large number of recommendations from different professionals are presented, which can be overlapping and contradictory. The program is responsible for coordinating these recommendations. At this time, the aim is to design an individualized care plan by establishing the goals and priorities of treatment and identifying the roles to be assumed by patients, caregivers, and professionals. To achieve this, the SPICA program searches for a common ground of understanding among professionals and the patient that allows the development of a care plan that matches the patient's preferences and is congruent with medical expertise and the best available evidence, but also feasible to apply in their environment.

In these special circumstances of the case and care complexity and taking into account that the patient is experiencing a stressful life event, shared decision-making is desirable but difficult. To reach a mutual decision, the SPICA team must gain the trust of the patient/caregiver. This trust is acquired when the patient, relative, and caregiver (depending on the case) have the perception that the professional has a deep understanding of the patient and by maintaining frequent contact during hospitalization. After exploring whether the patient and/or caregiver wants or can make decisions, the professional exposes the options. Once exposed, it is ensured that the patient and/or caregiver are able to understand them, and later, a decision will be made together. On occasions, it is advisable to have some time before making a decision. It should be noted that this plan is dynamic and interactive, and this is important for the patient to understand.

### Transferring the care plan

**Figure 1:**
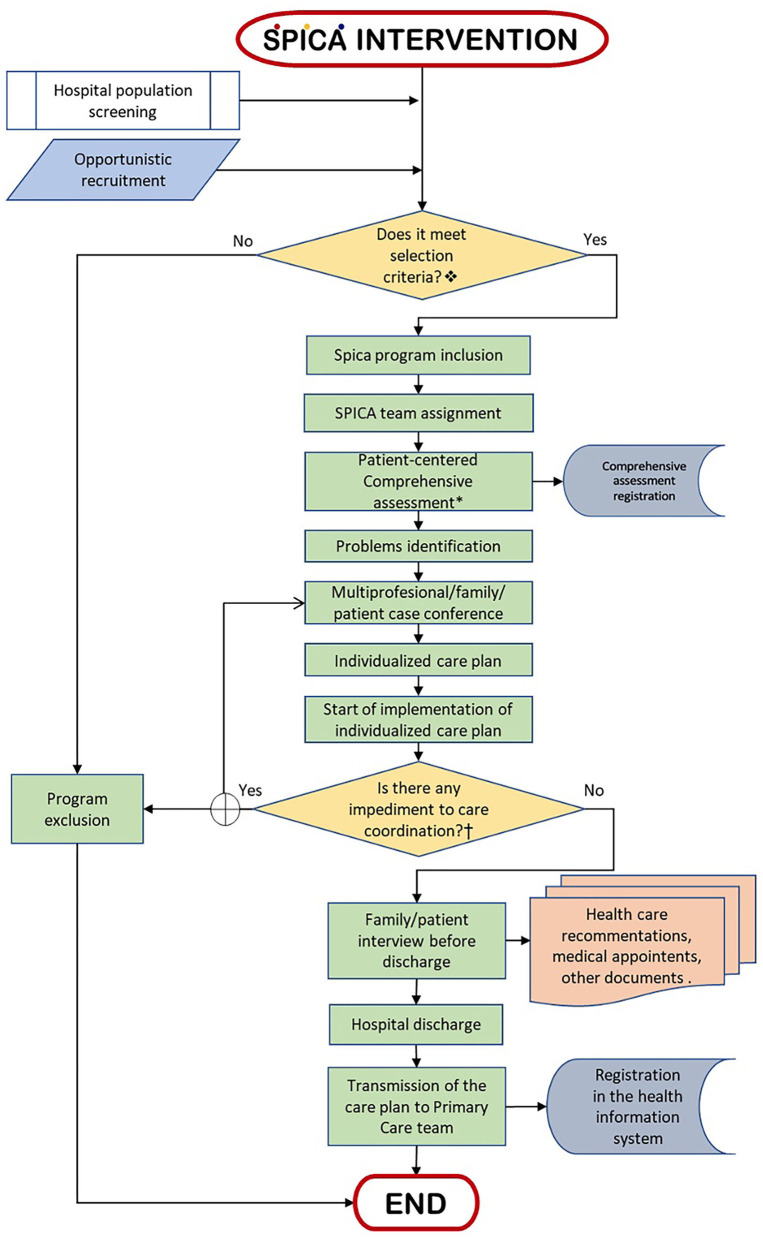
Flowchart (summarized) of the SPICA intervention process. 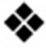
Inclusion criteria of the SPICA program are described in Table 1. *The sections that make up the SPICA comprehensive assessment are described in Supplementary Annex I.^†^Circumstances that do not allow the care coordination for patients included in SPICA program are: transfer of patients to other hospital or an intermediate care facility; death; or patient/family rejection.

**Figure 2:**
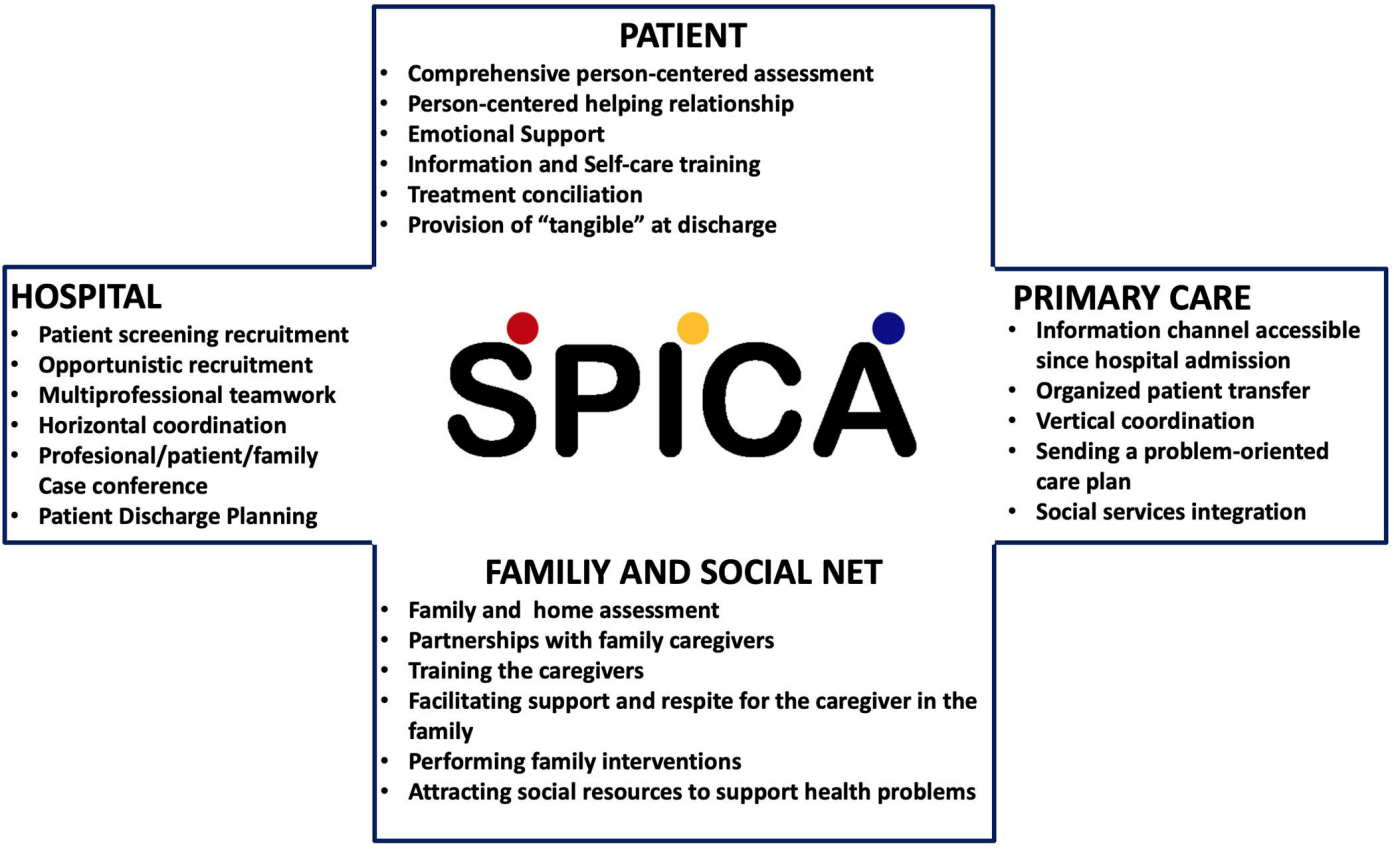
Main components of the Spica intervention.

During admission, continuous contact is maintained not only with the patient and their family/caregivers but also with their primary care providers, relaying a comprehensive report. At discharge, honoring that intention of maintaining continuity of care, the SPICA team is in charge of transmitting this information in the most detailed way possible, both to the professionals and to the patient/caregiver (refer to Figure 1 and Supplementary Appendix II). Moreover, the intervention seeks to improve the quality of life by enhancing the recovery of the patient's previous state of health and, if this is not possible, helping to accept and cope with the new health situation, promoting self-care training, empowering the patient, providing emotional support, and enhancing the patient-clinician relationship (refer to Figure 2).

## Discussion section that shares practical implications and lessons learned for future applications

**Figure 3:**
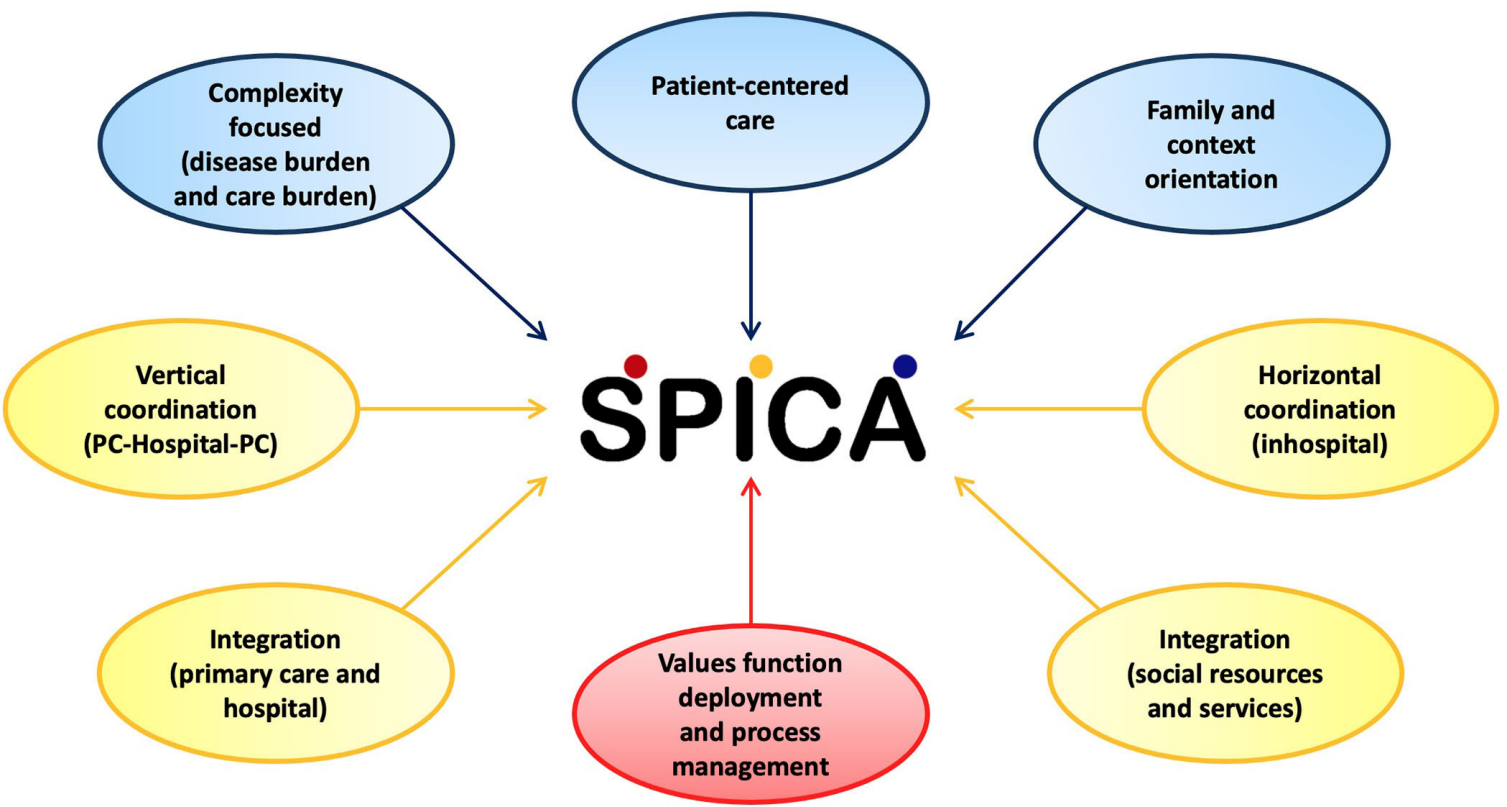
Major components of the Spica design (PC, primary care).

The fact that the SPICA program was proposed in 2000 in “La Laguna-Tenerife Norte” Multiprofessional Teaching Unit of Family and Community Care represents a singularity in the Spanish National Health Service. The team that makes up this Teaching Unit identifies itself with common professional values that try to make resident doctors and nurses visible during their training. That is what we call “values function deployment” (51). These values are Science, Humanity, Commitment, and Excellence. In this value-oriented training plan, it is created the conditions in which residents are exposed to the “daily experience of value,” as a necessary starting point for learning. The specialized training is based on supervised clinical practice and personal study, with the progressive assumption of responsibility, and is complemented by other types of regulated training activities. Regarding this commitment to training, the SPICA program is an ideal setting for learning fundamental values, knowledge, and abilities of the specialty of family and community care itself (refer to Figure 3). Every year, fifteen resident medical doctors in their third year of training and six resident nurses in their second year of training from this Teaching Unit are trained by working in this program. Each of them does it for 2 and 1.5 months, respectively (refer to Supplementary Appendices III, IV). In turn, this program receives resident nurses and doctors from other Teaching Units in Spain.

This program is committed to offering quality healthcare, which is why it pursues continuous quality management. Its first evaluation, at the end of 2000, showed positive effects in reducing hospital stays and readmissions. In later years, evaluations showed similar results and were presented at different national and regional congresses. Positive results have been observed in patient satisfaction. In addition, focus groups have been carried out to explore the perception of patients and caregivers, as well as primary care professionals, which has allowed us to know the utilities perceived by them and some improvement areas. The feedback from the different hospital services is collected through daily interaction and through the presentation of the annual results in joint sessions.

In 2006, a complete review of the program was carried out to adapt it to Process Management System and integrate it into the hospital and primary care management systems. Subsequently, in 2009, the program was reviewed again, to adapt to the ISO 9001 standard. The program was audited the same year, obtaining the certificate. Besides, in 2010, within the framework of management by processes, Hospital Universitario de Canarias defined the hospitalization and discharge process, and the SPICA program is embedded within it. In 2012 and 2013, improvements in interprofessional communication were incorporated. So, the SPICA program has been included in the hip fracture clinical pathway (since 2013) and the amyotrophic lateral sclerosis committee of Hospital Universitario de Canarias (since 2022). Although these improvements, the program's key components have remained stable since its formation in 2000.

Nowadays, the program is developing new research projects with the aim of reevaluating the effectiveness and cost-effectiveness of the program and its individualized care plans, focusing on the help relationship established with the patients. Thus, current interests also improve research on the creation and establishment of a common ground for understating meanings, problems, and/or conditions where shared decision-making occurs in vulnerable environments.

## Acknowledgment of any conceptual or methodological constraints

Some of the limitations that SPICA faces derive from the characteristics of the context that make it necessary.

Organizational culture understood as the set of predominant values, attitudes, and behaviors that characterize the functioning of an organization (52), is crucial in a health system. The Spanish Health Care System is focused mainly on single diseases, and clinical guidelines usually take a single-morbidity approach (53). This ignores the complexity in caring for the increasing number of patients with multimorbidity. The fact that our health organization has been eminently sectorized by diseases or medical specialties makes it difficult to change the perspective of health professionals toward a more holistic, comprehensive, and cooperative approach (54). In 2012, a chronicity management strategy was attempted in our country. However, this strategy has not been properly implemented and has not received the necessary resources. So, it has not had the desired success (55). These reflect the need for a change in the organizational culture that enhances primary care and its role in coordinating the global care of patients, regardless of where they are.

Family medicine in Spain is going through an identity crisis (56, 57), and primary care has organizational problems that prevent it from exercising its leadership as the axis of the system. In our environment, there is no notification system for hospital admissions and hospital discharges, which notifies the primary care professional of what is happening. The clinical history recording system is organized by episodes, which facilitates the care of acute patients but makes it difficult to carry out the longitudinal follow-up of chronic patients. Furthermore, its design does not prioritize continuity, affecting all its components: informative, longitudinal, and interpersonal (58, 59). This will have implications from an organizational and clinical perspective (50).

The integration of services is consistent from the point of view of financing, organization, provision of services, and clinical practices, to improve care for complex people. The health sectorization, which we have already mentioned, prevents this integration. The SPICA program depends on primary care management but assumes responsibility for planning the hospital discharge of the most complex patients. Besides, this is made without an integration structure or hierarchy. The stability of this functionality over time is based on an agreement between managers (hospital and primary care ones) that assumes a win-win negotiation and on the team's ability to adequately manage the soft power that has been granted to it.

Difficulties in integration occur not only among clinical areas but also between health and social ones. Although the experiences in countries such as the USA (60) and England (61) have been promising, the truth is that in our country, there is still a sectorization between social and clinical services that greatly limit efficient system development. In the SPICA program, professionals work as a team with social workers, despite these barriers, offering the patient the possibility of integrating both spheres.

Since its creation, the SPICA program has covered the entire reference population of a tertiary care hospital on the island of Tenerife. His career and permanence in time speak in favor of its usefulness, in addition to all the aspects reported in this article. Thereby, the program can be considered a singularity among the Family and Community Care Teaching Units in Spain. “La Laguna–Tenerife Norte” Teaching Unit has direct clinical responsibilities, and in this sense, it has a great similarity with any other hospital services. That is not the usual framework in which the Family and Community Care Teaching Units are structured in our environment (Spanish Health System). In fact, SPICA depends financially and organically on Primary Care but attends to the patients within the hospital, is integrated into the hospital's process map, is also part of their discharge planning program, and supports various clinical pathways of the hospital itself. These disruptive elements with respect to the care and teaching model established in Spain, which are considered key elements of its success, can also become barriers to entry or elements of difficulty to encourage other managers and Teaching Units to follow the same path. During all these years, the team has invested all their energy in fully developing the program by implementing systems of management, evaluation, and quality improvement. So, a process of external communication, sharing experience with the scientific, professional, and management community, is currently started through this study and successes that are in the pipeline.

## Data availability statement

The original contributions presented in the study are included in the article/Supplementary material, further inquiries can be directed to the corresponding author.

## Author contributions

MG-H and BG contributed to the conceptualization and writing and the editing. SB-C participated in the conceptualization and revision. JV-D participated in the conceptualization and writing and revision. All authors contributed to manuscript revision, read, and approved the submitted version.

## Conflict of interest

The authors declare that the research was conducted in the absence of any commercial or financial relationships that could be construed as a potential conflict of interest.

## Publisher's note

All claims expressed in this article are solely those of the authors and do not necessarily represent those of their affiliated organizations, or those of the publisher, the editors and the reviewers. Any product that may be evaluated in this article, or claim that may be made by its manufacturer, is not guaranteed or endorsed by the publisher.
