# Supplementary material for: Multicomponent, high-intensity, and patient-centered care intervention for complex patients in transitional care: SPICA program
Source: Front Med (Lausanne). 2022 Nov 24;9:1033689. doi: 10.3389/fmed.2022.1033689 (PMC9729702; doi:10.3389/fmed.2022.1033689)
Supplement: Supplementary file 1 [file Table_1.DOC]

| Clinical service: | | | | Room: |
| --- | --- | --- | --- | --- |
| Diagnosis on admission: | | | | |
| Admission date: | | | SPICA assessment date: | |
| Exclusionn | 🞎 | Exclusion cause: | | |

| Patient’s family doctor: | | Family unit clinic: |
| --- | --- | --- |
| Patient’s primary nurse: | SPICA doctor:  SPICA nurse: | |

· Risk Profile (Inclusion: 1 major or 3 minor criteria)·

| ***MAJOR CRITERIA*** | | Live alone |  | Previous diagnosis of depression |  |
| --- | --- | --- | --- | --- | --- |
| Previous home care |  | Included in spica program the previous year |  | Severe hearing or visual impariments |  |
| Terminal illness |  | ***MINOR CRITERIA*** |  | Malnutrition or morbid obesity |  |
| Cognitive impairment (Pf>4) |  | Age>74 |  | Admission in previous 6 months |  |
| Dependency for basic activities of daily living (Katz>A) |  | >2 Chronic deseases |  | Dependency for instrumental activities |  |
| Admission due to disabling fracture |  | Self-perceived poor health |  | Falls in previous 3 months |  |

**Active problems before admission:**

|  |  |
| --- | --- |

**Personal history:**

|  |  |
| --- | --- |

**· Cognitive assessment: Pfeiffer's SQMSQ test ·**

Pre-admission medication:

| **Does the patient know how to read and write?** | | **Yes** | **No** |
| --- | --- | --- | --- |
|  | | Hospitalization | Discharge |
| What are the date, month and year? | |  |  |
| What is the day of the week? | |  |  |
| What is the name of this place? | |  |  |
| What is your phone number? | |  |  |
| How old are you? | |  |  |
| When were you born? | |  |  |
| Who is the current president? | |  |  |
| Who was the president before him? | |  |  |
| What was your mother’s maiden name? | |  |  |
| Can you count backward from 20 by 3’s? | |  |  |
| *Success: A* | **Nº Errors** |  | |
| *Error: E* | **Correction** |  | |

PHYSICAL FUNCTION ASSESSMENT:

| KATZ INDEX | | **BATHING** | |  |  |  |  |  |
| --- | --- | --- | --- | --- | --- | --- | --- | --- |
| ❒ | No need assistance (patient gets in and out of the bathtub by himself, if this is his usual way of bathing) | ❒ | Need help cleaning only one part of the body (eg back and legs) | ❒ | Needs help with bathing morn tan one part of the body getting in or out of the tub or shower. Requires total bathing. | | | |
| **DRESSING** | | | | | |  |  |  |
| ❒ | Picks up clothes and dresses completely without help | ❒ | No help except to tie shoes | ❒ | Needs help with dressing self or needs to be completely dressed. | | | |
| **TOILETING** | | | | | |  |  |  |
| ❒ | Patient goes to the toilet, cleans himself, and adjusts his clothes without help (can use a cane, walker, or wheelchair). He can use the potty or bedpan at night, emptying it in the morning. | | | ❒ | Needs help transferring to the toilet, cleaning self or uses bedpan or commode. | | | |
| **TRANSFERRING** | | | | | |  |  |  |
| ❒ | Patient gets in and out of bed. Patient sits and stands without help (may use cane or walker) | | | ❒ | Needs help in moving from bed to chair or requires complete transfer. | | | |
| **CONTINENCE** | | | | | |  |  |  |
| ❒ | Fully control both sphincters | ❒ | Occasional incontinence | ❒ | Is partially or totally incontinent of bowel or bladder. | | | |
| **FEEDING** | | | | | |  |  |  |
| ❒ | No needs | ❒ | Need help only to cut the meat or spread the bread | ❒ | Needs partial or total help with feeding or requires parenteral feeding. | | | |
| **A:** Independent for all activities; B: Independent for all functions except one; C: Independent for all functions except bathing and one more; D: Independent for all functions except bathing, dressing, and one other; E: Independent for all functions except bathing, dressing, toileting and more; F: Independent for all functions except bathing, transferring and one more; G: Dependent for all functions; H: Dependent on at least 2 functions and not classifiable as C, D, E, or F. | | | | | |  |  |  |

| LAWTON - BRODY INSTRUMENTAL ACTIVITIES OF DAILY LIVING SCALE | | | **points** |
| --- | --- | --- | --- |
| ***Ability to Use Telephone***   - Operates telephone on own initiative-looks up and dials numbers, etc - Dials a few well-known numbers - Answers telephone but does not dial - Does not use telephone at all | | | 1  1  1  0 |
| ***Shopping***:   - Takes care of all shopping needs independently - Shops independently for small purchases - Needs to be accompanied on any shopping trip - Completely unable to shop | | | 1  0  0  0 |
| ***Food Preparation***   - Plans, prepares and serves adequate meals independently - Prepares adequate meals if supplied with ingredients - Heats, serves and prepares meals, or prepares meals, or prepares meals but does not maintain adequate diet - Needs to have meals prepared and served | | | 1  0  0  0 |
| ***Housekeeping***   - Maintains house alone or with occasional assistance (e.g. "heavy work domestic help") - Performs light daily tasks such as dish washing, bed making - Performs light daily tasks but cannot maintain acceptable level of cleanliness - Needs help with all home maintenance tasks - Does not participate in any housekeeping tasks | | | 1  1  1  1  0 |
| ***Laundry***   - Does personal laundry completely - Launders small items-rinses stockings, etc. - All laundry must be done by others | | | 1  1  0 |
| ***Mode of Transportation***   - Travels independently on public transportation or drives own car - Arranges own travel via taxi, but does not otherwise use public transportation - Travels on public transportation when accompanied by another - Travel limited to taxi or automobile with assistance of another - Does not travel at all | | | 1  1  1  0  0 |
| ***Responsibility for Own Medications***   - Is responsible for taking medication in correct dosages at correct time - Takes responsibility if medication is prepared in advance in separate dosage - Is not capable of dispensing own medication | | | 1  0  0 |
| ***Ability to Handle Finances***   - Manages financial matters independently (budgets, writes checks, pays rent, bills, goes to bank), collects and keeps track of income - Manages day-to-day purchases, but needs help with banking, major purchases, etc. - Incapable of handling money | | | 1  1  0 |
| **CORRECTION IN MEN:** |  | **Total** |  |

**Self-perceived health:**

How do you consider your health compared to those of your age?

| Excelent | Very good | good | poor | bad | Very bad |
| --- | --- | --- | --- | --- | --- |

**Illness experience and psychoaffective assessment:**

| **HEARING ASSESSMENT** | **VISUAL ASSESSMENT** |
| --- | --- |
| 1. **Hears adequately** 2. **Minimum difficulty:** does not hear when there is no silence 3. **Hears only in special situations:** need to increase voice tone, speak slowly 4. **Very limited:** no hearing | **0. Adequate:** sees print from newspaper or book  **1. Slightly altered:** headline font, does not read text  **2. Moderately impaired:** does not see headlines, identifies objects  **3. Very disturbed:** identifies objects with difficulty; eyes seem to follow objects  **4. Severely impaired:** does not see or sees only light, colors, or shapes; the eyes do not follow objects. |
| Does the patient use hearing aids? ❒ Yes ❒ No | Does the patient use visual aids? ❒ Yes ❒ No |
| **Severe hearing decifit > 1** ❒ Yes ❒ No | **Severe visual deficit > 2** ❒ Yes ❒ No |

**NUTRITIONAL ASSESSMENT:**

| **Subjective Assessment** | **Severe muscle wasting or fat loss** | **Moderate muscle wasting or fat loss** | **No atrophy or loss** |
| --- | --- | --- | --- |
| **Upper limbs** | **2** | **1** | **0** |
| **Lower limbs** | **2** | **1** | **0** |
| **temporalis muscle** | **2** | **1** | **0** |
| **bichat ball** | **2** | **1** | **0** |
| **Abdominal subcutaneous fat** | **2** | **1** | **0** |

Poor prognosis > 4 points.

• Weight and/or BMI:

• Appetite:

• State of dentition:

• Chewing and swallowing:

• Elimination:

**Skin condition:**

**RISK OF FALLS:**

|  | Previous falls (last year) |  | Dependent for basic activities of daily living |
| --- | --- | --- | --- |
|  | Walking disorder |  | Behavior disorders/agitation |
|  | Parkinson's disease |  | Hypotensive treatment |
|  | Stroke with motor sequelae |  | Antiarrhythmic treatment |
|  | Peripheral neuropathy |  | Anxiolytic and/or hypnotic treatment |
|  | Significant lower limb osteoarthritis |  | Antidepressant treatment |
|  | Chronic dizziness or vertigo |  | Neuroleptic treatment |
|  | Orthostatic hypotension |  | Antiepileptic treatment |
|  | Visual deficit |  | Home barriers |
|  | Cognitive impairment |  |  |

**Notes:**

**KIND OF FAMILY:**

- 1- Nuclear
- 2- Binuclear
- 3- Single parent
- 4- Family equivalents
- 6- Live alone
- 4- Extensive

| **FEATURES:** | Yes | No |
| --- | --- | --- |
| Enlarged |  |  |
| Numerous |  |  |
| with close relatives |  |  |

**FAMILY LIFE CYCLE:**

- 1- Home abandonment
- 2- Couple formation
- 3- Pregnancy and childbirth
- 4- Family with small children
- 5- Family with teeagers
- 6- Young and middle-aged adult life
- 7- Family contraction
- 8- Death and mourning

**NUMBER OF COHABITANTS: _____ Who are they?**

- 1- Spouse __
- 2- Sons __
- 3- Daughters __
- 4- Grandchildren __
- 5- Sons-in-law __
- 6- Daughters-in-law__
- 7- Brothers__
- 8- Sisters __
- 9- Nephews __
- 10- Other relatives __
- 11- Others_

**CONFLICTIVE RELATIONSHIPS IN THE FAMILY CORE**: 🞎 Yes 🞎 No

**LIFE EVENTS (last year):**

- 1- Spouse death
- 2- Divorce or marital separation
- 3- Close relative death
- 4- Personal injury or illness
- 5- Retirement and/or change of economic situation
- 6- Family member illness
- 7- Sexual problemss
- 8- Adding a new member to the family
- 9- Death of a close friend
- 10- Poor marital relationship
- 11- Child who leaves home
- 12- Change of residence
- 13- Others

| **DID THE PATIENT NEED A CAREGIVER BEFORE ADMISSION?**  🞎 Yes 🞎 No  If yes, who or whom?:  Name:__________________________   - 1- Spouse - 2- Son/Daughter - 3- Grandson - 4- Son-in-law/Daughter-in-law - 5- Brother/sister - 6- Nephew - 7- Other relative - 8- No relative   Does the caregiver live at the same address? 🞎 Yes 🞎 No  If not, Telephone_____________________  ________________________________ | **WILL THE PATIENT NEED A CAREGIVER AFTER DISCHARGE?**  🞎 Yes 🞎 No  If yes, who or whom?:  Name:__________________________   - 1- Spouse - 2- Son/Daughter - 3- Grandson - 4- Son-in-law/Daughter-in-law - 5- Brother/sister - 6- Nephew - 7- Other relative - 8- No relative   Does the caregiver live at the same address? 🞎 Yes 🞎 No  If not, Telephone_____________________ |
| --- | --- |

| **APGAR FAMILIAR:** | **Hardly ever** | **Some of the time** | **Almost always** |
| --- | --- | --- | --- |
| Can you turn to your family for help when something is troubling you? | 0 | 1 | 2 |
| Are you satisfied with the way your family talks things over with you and shares problems with you? | 0 | 1 | 2 |
| Are you satisfied that your family accepts and supports you wishes to take on new activities or directions? | 0 | 1 | 2 |
| Are you satisfied with the way your family expresses affection and responds to your emotions, such as anger, or love? | 0 | 1 | 2 |
| Are you satisfied with the way your family share time together with you? | 0 | 1 | 2 |

Genogram: Date ______________

House characteristics:

Patient resources:

**GIJÓN SOCIAL ASSESSMENT SCALE SHORT VERSION GIJÓN SOCIAL ASSESSMENT SCALE**

| **Family situation** | |
| --- | --- |
| 1. | Live with a partner and/or family without conflict |
| 2. | Live with a partner of similar age |
| 3. | Lives with a partner and/or family and/or others, but they cannot or do not want to care for the patient |
| 4. | Live alone. Sons/Daughters or close relatives who do not cover all the needs. |
| 5. | Live alone. Distant family or no family. |
| **Social relationships and contacts** | |
| 1. | Maintains social relationships outside home. |
| 2. | Only relates to family, neighbors, or others. Leaves home. |
| 3. | Only relates to family. Leaves home. |
| 4. | Does not leave his home. Receives family or visitors (> 1 per week). |
| 5. | Does not leave his home. Receives visits (< 1 per week) |
| **Social network supports** | |
| 1. | Needs no support. |
| 2. | Receives support from family and/or neighbors. |
| 3. | Receives sufficient formal social support (care center, family worker, lives in residence...). |
| 4. | Has social support but it is insufficient. |
| 5. | Hasn’t got any social support and needs it. |

| **Family situation** | |
| --- | --- |
| 1. | Live with a partner and/or family without conflict |
| 2. | Live with a partner of similar age |
| 3. | Lives with a partner and/or family and/or others, but they cannot or do not want to care for the patient |
| 4. | Live alone. Sons/Daughters or close relatives who do not cover all the needs. |
| 5. | Live alone. Distant family or no family. |
| **Economic situation** | |
| 1. | >1,5 times the minimum salary |
| 2. | 1,5 times the minimum salary up to the minimum salary |
| 3. | Minimum salary up to minimum contributory pension |
| 4. | No contributory pension |
| 5. | Lower income or no income |
| **Social relationships and contacts** | |
| 1. | Maintains social relationships outside home. |
| 2. | Only relates to family, neighbors, or others. Leaves home. |
| 3. | Only relates to family. Leaves home. |
| 4. | Does not leave his home. Receives family or visitors (> 1 per week). |
| 5. | Does not leave his home. Receives visits (< 1 per week) |
| **Social network supports** | |
| 1. | Needs no support. |
| 2. | Receives support from family and/or neighbors. |
| 3. | Receives sufficient formal social support (care center, family worker, lives in residence...). |
| 4. | Has social support but it is insufficient. |
| 5. | Hasn’t got any social support and needs it. |

**Short scale:** >=10 points: High social risk, high risk of institutionalization

**Original version:** >=17 points: High social risk

| Problem | Aims | TASK | Roles |
| --- | --- | --- | --- |
|  |  |  |  |
|  |  |  |  |
|  |  |  |  |
|  |  |  |  |
|  |  |  |  |
|  |  |  |  |

| **CHECK LIST OF PREVIOUS TASKS AND AT THE TIME OF HOSPITAL DISCHARGE** | **Yes** | **No** |
| --- | --- | --- |
| Has the hospital discharge date been agreed upon with the responsible doctor, patient and/or family/caregiver? |  |  |
| Has the patient and/or family/caregiver been informed of the hospital discharge date? |  |  |
| Has the comprehensive assessment been carried out prior to hospital discharge? |  |  |
| Has the pre-discharge clinical situation been reviewed (analytics, constants, catheters, symptoms and warning signs)? |  |  |
| Has the condition of the skin and the surgical wound(s) been reviewed? |  |  |
| Do I have a clearly established list of problems with which to work on hospital discharge? |  |  |
| Have I updated and reconciled the therapeutic plan in agreement with the responsible doctor? |  |  |
| Has the patient and/or family/caregiver been given a printed copy of prescription? |  |  |
| Have graphic explanations of updated treatment been provided to the patient/family/caregiver? |  |  |
| Does the patient carry any prescription that requires the approval of medical inspection? |  |  |
| If the medical inspection approval is required (drugs, diapers, nutritional supplements), has it been processed? |  |  |
| Does the patient have enough medication until the medical inspector authorizes that prescription? |  |  |
| If you need hospital pharmacy drugs, have they been collected? |  |  |
| If you need some type of orthosis, has it been processed? |  |  |
| Does the patient have the necessary medical reports to request social assistance resources? |  |  |
| Have appointments been delivered to the patient with primary and hospital care? |  |  |
| If the appointment is at home, has it been agreed with the primary care professional? |  |  |
| Are the dates of the appointments that were managed recorded in the Global Discharge Care Plan? |  |  |
| Was the patient included in the Drago-AP History Service Portfolio Code 900? |  |  |
| If the patient requires non-urgent medical transport on the day of discharge, has the request been coordinated? |  |  |
| If the patient needs medical transport for appointments after discharge, have you managed or informed about the procedures? |  |  |
