# Supplementary figures and images for: Multicomponent, high-intensity, and patient-centered care intervention for complex patients in transitional care: SPICA program
Source: Front Med (Lausanne). 2022 Nov 24;9:1033689. doi: 10.3389/fmed.2022.1033689 (PMC9729702; doi:10.3389/fmed.2022.1033689)

## Appendix II.- Complete flowchart of the SPICA process

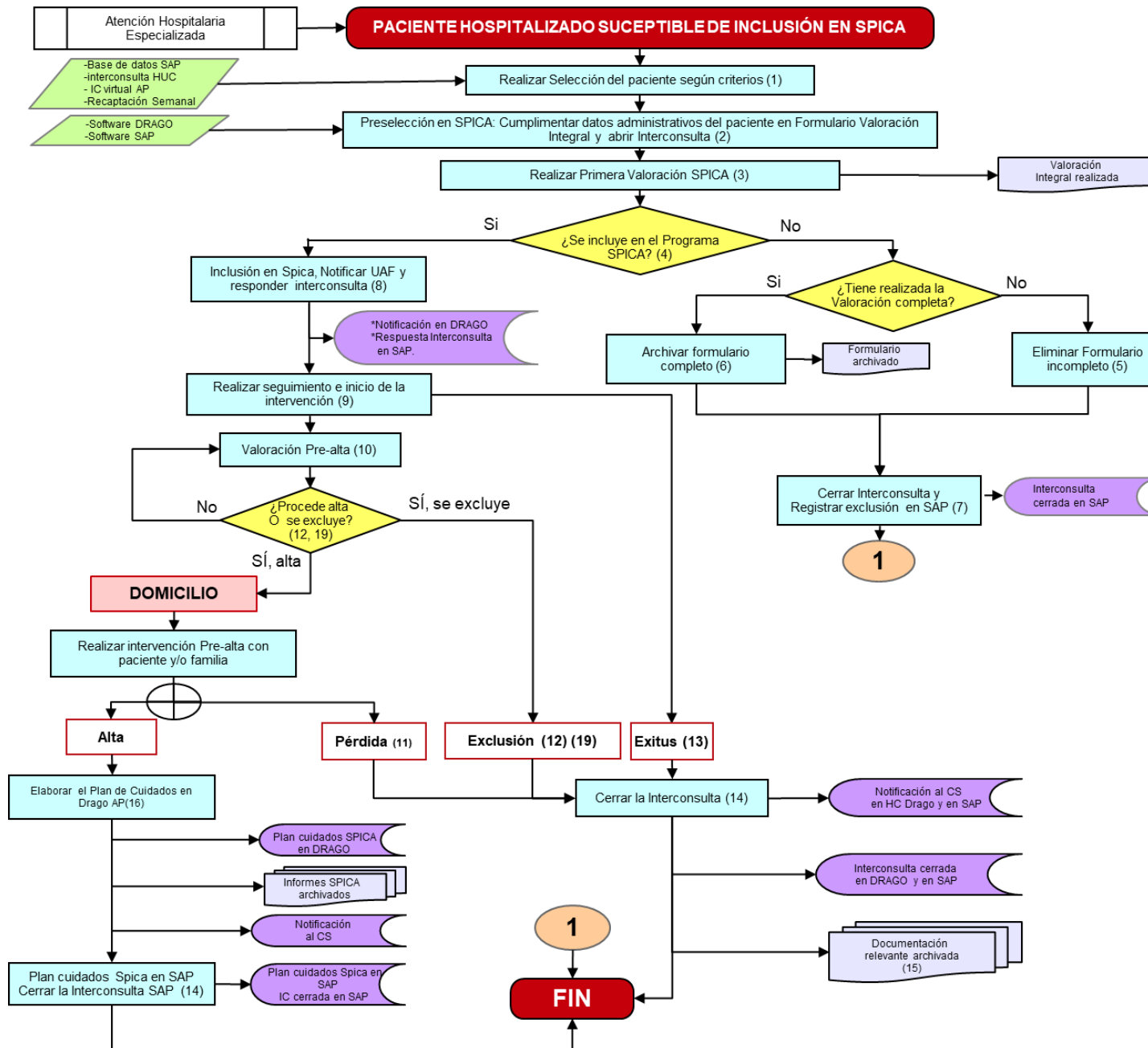

Supplement: Supplementary file 2 [file Data_Sheet_1.PDF]
